# Performance Deficits of NK1 Receptor Knockout Mice in the 5-Choice Serial Reaction-Time Task: Effects of *d*-Amphetamine, Stress and Time of Day

**DOI:** 10.1371/journal.pone.0017586

**Published:** 2011-03-07

**Authors:** Ting Carrie Yan, Julia A. Dudley, Ruth K. Weir, Ewelina M. Grabowska, Yolanda Peña-Oliver, Tamzin L. Ripley, Stephen P. Hunt, David N. Stephens, S. Clare Stanford

**Affiliations:** 1 Department of Neuroscience, Physiology and Pharmacology, University College London, London, United Kingdom; 2 Department of Cell and Developmental Biology, University College London, London, United Kingdom; 3 School of Psychology, University of Sussex, Falmer, Brighton, United Kingdom; Duke University, United States of America

## Abstract

**Background:**

The neurochemical status and hyperactivity of mice lacking functional substance P-preferring NK1 receptors (NK1R-/-) resemble abnormalities in Attention Deficit Hyperactivity Disorder (ADHD). Here we tested whether NK1R-/- mice express other core features of ADHD (impulsivity and inattentiveness) and, if so, whether they are diminished by *d*-amphetamine, as in ADHD. Prompted by evidence that circadian rhythms are disrupted in ADHD, we also compared the performance of mice that were trained and tested in the morning or afternoon.

**Methods and Results:**

The 5-Choice Serial Reaction-Time Task (5-CSRTT) was used to evaluate the cognitive performance of NK1R-/- mice and their wildtypes. After training, animals were tested using a long (LITI) and a variable (VITI) inter-trial interval: these tests were carried out with, and without, *d*-amphetamine pretreatment (0.3 or 1 mg/kg i.p.). NK1R-/- mice expressed greater *omissions* (inattentiveness), *perseveration* and *premature responses* (impulsivity) in the 5-CSRTT. In NK1R-/- mice, *perseveration* in the LITI was increased by injection-stress but reduced by *d*-amphetamine. *Omissions* by NK1R-/- mice in the VITI were unaffected by *d*-amphetamine, but *premature responses* were exacerbated by this psychostimulant. *Omissions* in the VITI were higher, overall, in the morning than the afternoon but, in the LITI, *premature responses* of NK1R-/- mice were higher in the afternoon than the morning.

**Conclusion:**

In addition to locomotor hyperactivity, NK1R-/- mice express inattentiveness, perseveration and impulsivity in the 5-CSRTT, thereby matching core criteria for a model of ADHD. Because *d*-amphetamine reduced *perseveration* in NK1R-/- mice, this action does not require functional NK1R. However, the lack of any improvement of *omissions* and *premature responses* in NK1R-/- mice given *d*-amphetamine suggests that beneficial effects of this psychostimulant in other rodent models, and ADHD patients, need functional NK1R. Finally, our results reveal experimental variables (stimulus parameters, stress and time of day) that could influence translational studies.

## Introduction

Attention Deficit Hyperactivity Disorder (ADHD) is a heritable, developmental disorder that affects between 2–5% of children in the UK but is prevalent worldwide [1]. Its core diagnostic features are hyperactivity, inattentiveness and impulsivity. The prominence and combination of these abnormalities define the diagnostic subtype, *viz*: Predominantly Inattentive, Predominantly Hyperactive/Impulsive or Combined Type [2]. Perseveration is also common in this disorder [3] but is not a diagnostic criterion.

Only three compounds are licensed to treat ADHD in the UK (*d*-amphetamine, methylphenidate and atomoxetine) [4] but guanfacine and the prodrug, lisdexamfetamine, are also available in the USA [5]. All these compounds augment monoamine transmission in the brain and periphery. However, their predictable hemodynamic side-effects, the unease about long-term use of the psychostimulants, *d*-amphetamine and methylphenidate (especially in children), and their lack of efficacy in approximately 20–25% of patients (*e.g*., [6]), justify the need for a better understanding of the neurobiological abnormalities underlying ADHD and development of alternative drug treatments.

Mice lacking functional substance P-preferring, neurokinin-1 (NK1) receptors, through either functional ablation of the tachykinin-1 receptor (*tacr1*) gene (‘NK1R-/-’, [7) or receptor antagonism, display locomotor hyperactivity that is prevented by *d*-amphetamine or methylphenidate [8], [9], [10]. There are also striking abnormalities in the regulation of noradrenergic [8], [9] dopaminergic [10], [11] and serotonergic [12] transmission in the prefrontal cortex and dorsal striatum of NK1R-/- mice. All these findings are consistent with evidence for dysfunctional corticostriatal brain circuits in ADHD (e.g., [13]). Our proposal that NK1R-/- mice offer a mouse model of this disorder [11] is further supported by the identification of disease susceptibility haplotypes in the human *tacr1* gene of patients with ADHD [10], [14].

Here, we investigated whether NK1R-/- mice also display inattentiveness and impulsivity. We compared their behavior with that of wildtypes in the 5-Choice Serial Reaction-Time Task (‘5-CSRTT’), which enables evaluation of several aspects of animals' cognitive performance and response control [15], [16], [17]. These include: *premature responses* (an index of one type of impulsivity (see: [18])) and *perseveration*, as well as *% incorrect responses* and *% omissions* (failure to respond in the task), both of which indicate inattentiveness.

After training the animals to criterion, they were tested under conditions that increased attentional demand in two different ways. The first prolonged the inter-trial interval (7 s: ‘LITI’) during which animals were required to withhold their motor response. The second used a randomised, variable inter-trial interval (2–15 s: ‘VITI’). Both procedures increase measures of *inattentiveness* and *premature responding* (see: [19]), but the latter prevents the time elapsed since the start of the trial from serving as a cue that would confound measures of animals' performance. We then went on to investigate whether any deficits in cognitive performance and response control in either of these tests are ameliorated by *d-*amphetamine.

Finally, there is a great deal of evidence linking disruption of circadian rhythms with ADHD. For instance, there are reports of: a polymorphism in the circadian gene, *CLOCK* [20]; disruption of sleep rhythms (*e.g.,* [21]) and fluctuation of inattentiveness with time of day [22] in ADHD patients. NK1R are prevalent in the rat intergeniculate leaflet, an area implicated in circadian control, and in the dorsolateral margin of the suprachiasmatic nucleus [23], which has an undisputed role in regulation of circadian rhythms. Furthermore, the NK1R antagonist, aprepitant (used clinically as an anti-emetic), can cause daytime fatigue and insomnia in humans, while another NK1R antagonist, GR 205 171, disrupts circadian rhythms of motor activity in rodents [24]. Prompted by all this evidence, the experimental design enabled us to investigate whether the performance of NK1R-/- and wildtype mice in the 5-CSRTT is influenced by the time of day during which the mice are trained and tested.

## Results

### Training

We compared the behavior of the two genotypes in two batches of mice (Cohort 1 and Cohort 2: see Methods). Because no differences between the two cohorts emerged, the data were pooled for evaluation of the main effects of genotype and time of day.

**Figure 1:**
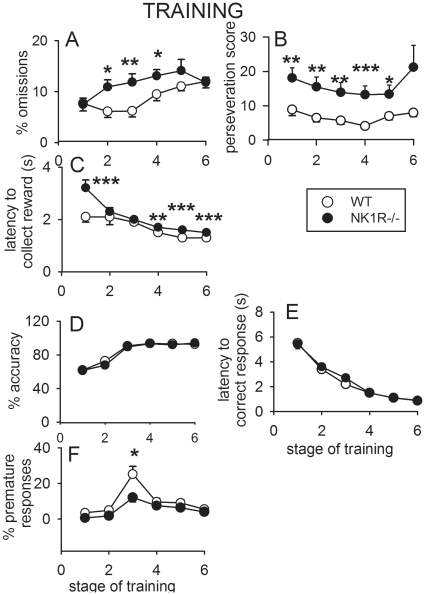
The performance of wildtype and NK1R-/- mice during training in the 5-CSRTT. *% Omissions*, *perseveration* and *latency to collect the reward* are all greater in NK1R-/- mice than wildtypes, regardless of time of day (A–C). There was no difference in *accuracy* (D) or *latency to correct response* (E), but premature responses (F) were greater in wildtypes, especially during Stage 3 of training. Points show mean ± s.e.m. * *P*<0.05, ** *P*<0.01, *** *P*<0.001. N = 23–24 per group.

*% Omissions* (F_(1,39)_ = 8.2, *P*<0.01) and *perseveration* (F_(1,39)_ = 23.3, *P*<0.001) were greater in NK1R-/- mice than wildtypes (Fig. 1A & 1B). *Latency to collect the reward* was also slightly greater in the knockouts (F_(1,39)_ = 22.8, *P*<0.001) (Fig. 1C). *Accuracy* and *latency to correct response* were not affected by genotype (Figs. 1D & 1E). Paradoxically, the incidence of *premature responses* across Stages 1–6 was greater in wildtype mice than NK1R-/- mice, overall (F_(1,43)_ = 11.5, *P*<0.001) (Fig. 1F) and increased transiently in both genotypes during Stage 3 of training, as has been reported previously [19].

The number of sessions needed for the mice to match the baseline criteria for testing depended on genotype. NK1R-/- mice needed more (*c*.15%) training sessions than wildtypes, overall (F_(1,43)_ = 4.14, *P*<0.05), but this depended on time of day to some extent (see below).

### Impaired cognitive performance of NK1R-/- mice tested with a long inter-trial interval (LITI)

**Figure 2:**
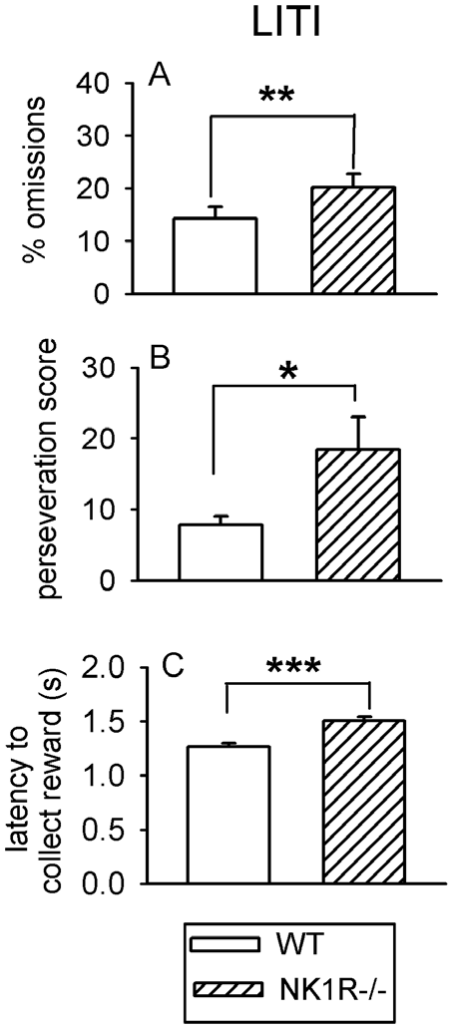
The performance of wildtype and NK1R-/- mice tested with a long ITI (‘LITI’) in the 5-CSRTT. *% Omissions* (A), *perseveration score* (B) and *latency to collect the reward* (C) are all greater in NK1R-/- mice than wildtypes, regardless of time of day, when tested with a long inter-trial interval (LITI). Bars show mean ± s.e.m score for each behavior. N = 23–24 per group. * *P*<0.05, ** *P*<0.01, *** *P*<0.001.

Again, there were no differences in the performance of mice from Cohort 1 and Cohort 2 and so the data were pooled for evaluation of the main effects of genotype and time of day. When tested with the LITI, *% omissions* (F_(1,43)_ = 7.63, P<0.01), *perseveration* (F_(1,43)_ = 5.41, *P*<0.05) and *latency to collect the reward* (F_(1,43)_ = 27.1, *P*<0.001) were all greater in NK1R-/- mice than the wildtypes (Fig. 2A–C). Other behavioral measures did not differ in the two genotypes (data not shown).

### Impaired cognitive performance of NK1R-/- mice tested with a variable inter-trial interval (VITI)

The overall incidence of certain behaviors differed in the two cohorts when tested in the VITI (Table 1). However, no behavior was influenced by an interaction between Cohort and either genotype or time of day and so the data from the two cohorts were pooled for statistical analysis of the main effects of these two factors.

**Table 1:** Behavior of the two cohorts of untreated mice in the VITI test.

		Wildtype	NK1R-/-
***% Accuracy***	Cohort 1 **	98.0±0.5	95.1±1.1
	Cohort 2	94.7±0.9	90.9±1.6
***Perseveration score***	Cohort 1 *	13.3±2.8	18.7±3.8
	Cohort 2	4.7±1.0	20.7±7.9
***% Premature responses***	Cohort 1 **	16.0±2.5	25.2±3.6
	Cohort 2	21.2±2.7	38.5±4.9

Animals' *accuracy, perseveration* and *premature responses* in the VITI test differed in the two cohorts, but there was no *interaction* between ‘cohort’ and ‘genotype’ or ‘time of day’ for any of these behaviors. N = 23–24 per group.

* *P*<0.05, ** *P*<0.01 (*c.f.,* Cohorts 1 and 2).

**Figure 3:**
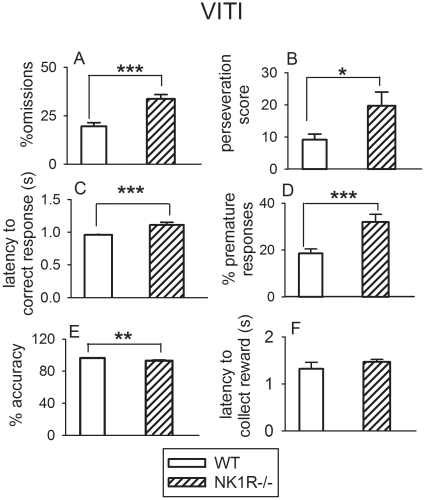
The performance of wildtype and NK1R-/- mice tested with a variable ITI (‘VITI’) in the 5-CSRTT. NK1R-/- mice show greater *% omissions* (A), *perseveration score* (B) *latency to correct response* (C), *% premature responses* (D) but lower *% accuracy* (E) than wildtypes and no difference in *latency to collect the reward* (F), regardless of time of day, when tested with a variable inter-trial interval (VITI). Bars show mean ± s.e.m for each behavioral score. * *P*<0.05, ** *P*<0.01, *** *P*<0.001 for differences between group means. N = 23–24 per group.

*% Omissions* were higher overall in NK1R-/- mice than wildtypes (F_(1,43)_ = 24.59, *P* = 0.001), as were *perseveration* (F_(1,43)_ = 4.95; *P*<0.05) and *latency to correct response* (F_(1,43)_ = 13.0, *P*<0.001) (Fig. 3A–C). The % *premature responses* was also greater in NK1R-/- mice (F_(1,39)_ = 14.9, *P*<0.001) (Fig. 3D), especially with the longer ITIs (*c.f.* wildtypes at 10 s and 15 s (*post hoc* tests): *P*<0.001 and *P*<0.05, respectively). *Accuracy* was also impaired in NK1R-/- mice, albeit to a small extent (3%: F_(1,39)_ = 7.96, *P*<0.01; Fig. 3E). There was no genotype difference in *latency to collect the reward* (Fig. 3F).

### Saline injection and *d*-amphetamine modify behavior in the 5-CSRTT

**Figure 4:**
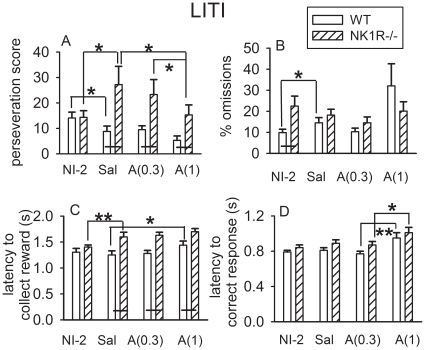
Effects of *d-*amphetamine on the behavior of wildtype and NK1R-/- mice tested with the LITI in the 5-CSRTT. The *perseveration score* of NK1R-/- mice is exacerbated by saline injection but ameliorated by *d-*amphetamine: the latter has no effect in the wildtypes (A). *d*-Amphetamine has no effect on *% omissions* in either genotype (B) but increases *latency to reward* in wildtypes (C) and *latency to correct response* (D) in both genotypes. Bars show mean ± s.e.m for the behavior of either untreated mice, tested for the second time with the LITI (NI-2), or mice given an i.p. injection of saline (Sal) or *d*-amphetamine (0.3 mg/kg, ‘A(0.3)’ or 1 mg/kg, ‘A(1)’). The mice experienced each treatment, once only, at weekly intervals. The sequence of treatments (including NI-2) was pseudo-randomised (latin-square) across the subjects. The black line linking adjacent bars indicates a genotype difference, regardless of time of day, of *P*<0.05, at least. N = 12 per group. * *P*<0.05, ** *P*<0.01, *** *P*<0.001 for comparisons of group means indicated above the bars.

In the LITI, *perseveration* was the only behavioral abnormality expressed by NK1R-/- mice to be ameliorated by *d*-amphetamine (Fig. 4A). Specifically, *d*-amphetamine restored baseline performance by preventing an increase in *perseveration* in NK1R-/- mice following an i.p. injection (*c.f., d*-amphetamine and saline: F_(2,34)_ = 5.5, *P*<0.05). This pattern of changes differed strikingly from that in wildtypes in which *perseveration* was reduced by an i.p. injection (*c.f.,* saline and NI-2: F_(1,22)_ = 9.8, *P*<0.01, t_11_ = 2.6, *P*<0.05) and unaffected by *d-*amphetamine.

Saline injection increased *% omissions* in wildtypes but did not affect NK1R-/- mice and so abolished the genotype difference seen in uninjected subjects (*c.f.,* saline and NI-2: F_(1,22)_ = 4.7, *P*<0.05; Fig. 4B). *d*-Amphetamine did not reduce *% omissions* in either genotype (Fig. 4B). *Latency to collect the reward* was increased by saline injection in NK1R-/- mice but unaffected by *d*-amphetamine whereas, in wildtypes, the opposite occurred: this behavior was unaffected by saline and increased by the higher dose of *d*-amphetamine (Fig. 4C). The *latency to correct response* was not affected by saline injection in either genotype but was increased by the higher dose of *d-*amphetamine in both (Fig. 4D). Neither saline nor *d-*amphetamine had any effect on *accuracy* or *premature responses* (data not shown).

**Figure 5:**
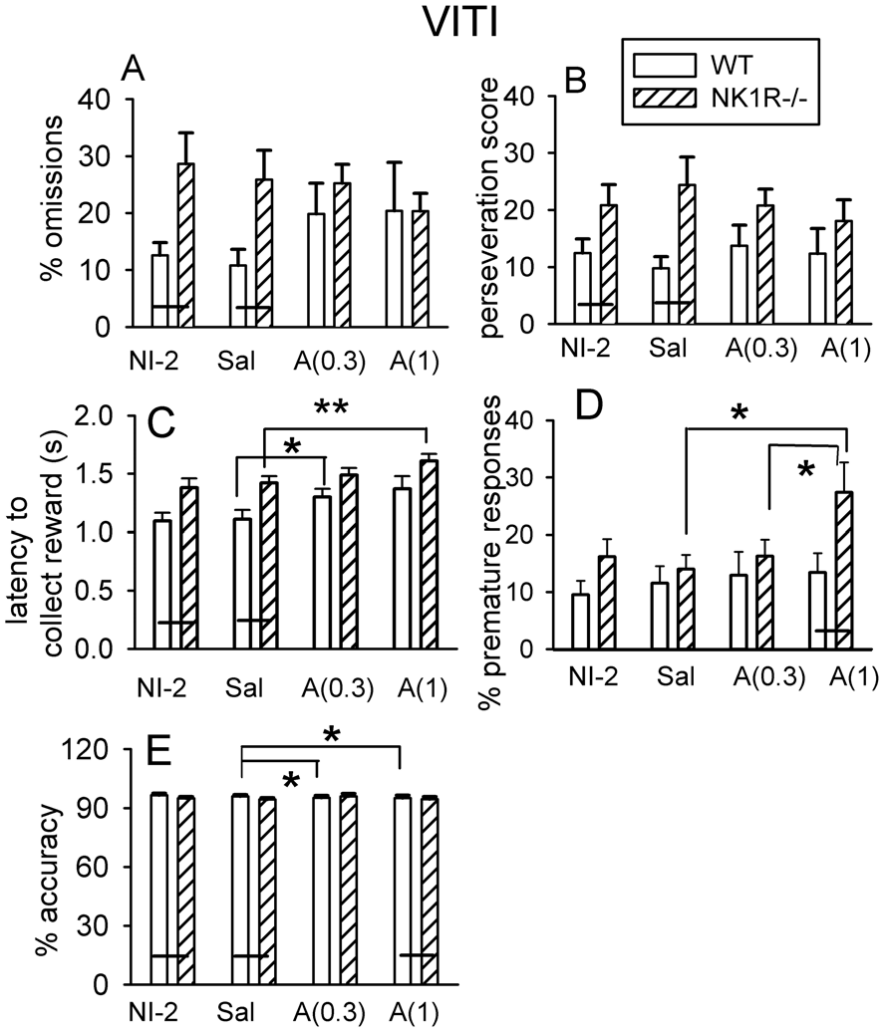
Effects of *d*-amphetamine on the behavior of wildtype and NK1R-/- mice tested with the VITI in the 5-CSRTT. *d*-Amphetamine did not affect *% omissions* (A) or *perseveration score* (B) but increased *latency to collect the reward* in both genotypes (C) and *% premature responses* in NK1R-/- mice (D). *d*-Amphetamine slightly reduced the *% accuracy* of wildtypes, only (E). Bars show mean ± s.e.m for the behavior of either untreated mice, tested for the second time with the VITI (NI-2), or mice given an i.p. injection of saline (Sal) or *d*-amphetamine (0.3 mg/kg, ‘A(0.3)’ or 1 mg/kg, ‘A(1)’). The mice experienced each treatment once only, at weekly intervals. The sequence of treatments (including NI-2) was pseudo-randomised (Latin-square) across the subjects. The black line linking adjacent bars indicates a genotype difference of *P*<0.05, at least, regardless of time of day. N = 11-12 per group. * *P*<0.05, ** *P*<0.01, *** *P*<0.001 of comparisons of groups means indicated above the bars.

In the VITI, saline injection did not affect any behavioral measure in either genotype whereas both doses of *d-*amphetamine abolished the genotype differences in *% omissions* (Fig. 5A), *perseveration* (Fig. 5B) and *latency to collect the reward* (Fig. 5C). These effects were a consequence of drug-induced changes in both genotypes (a reduction in NK1R-/- mice and an increase in wildtypes), rather than a selective action in NK1R-/- mice, but there was no statistically significant interaction between drug treatment and genotype. The higher dose of *d*-amphetamine actually increased *premature responses* in NK1R-/- mice (F_(2,42)_ = 3.6, *P*<0.05) (Fig. 5D) and slightly reduced the *accuracy* of wildtypes (Fig. 5E). There were no drug effects on *latency to correct response* (data not shown).

### Circadian influences on behavior

**Figure 6:**
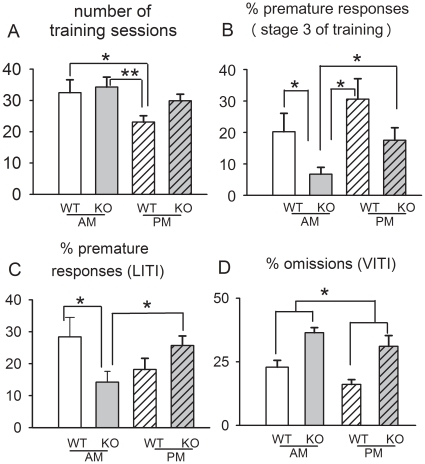
Behaviors of NK1R-/- and wildtype mice in the 5-CSRTT that depend on time of day. (A) the number of sessions needed to train mice to baseline criteria for testing; (B) *% premature responses* during stage 3 of training; (C) *% premature responses* in the LITI test; (D) *% omissions* in the VITI test. Bars show mean ± s.e.m. * *P*<0.05, ** *P*<0.01 for comparisons indicated. N = 23–24 per group.

Several aspects of animals' behavior depended on time of day. During training, wildtypes and NK1R-/- mice trained in the morning needed more sessions to stabilize at the baseline criterion for testing than did wildtypes trained in the afternoon (F_(1,43)_ = 5.7, *P*<0.05) (Fig. 6A). Moreover, *premature responses* during Stage 3 were lower in NK1R-/- mice trained in the morning than all other groups (F_(1,43)_ = 16.5, *P*<0.001) (Fig. 6B).

In the LITI, *premature responses* were influenced by an interaction between genotype and time of day (F_(1,43)_ = 6.6, *P*<0.05): their incidence in the NK1R-/- group that were tested in the morning was only 36% of that in wildtypes but, in the afternoon, this behavior increased in NK1R-/- mice and no longer differed in the two genotypes (Fig. 6C).

*% Omissions* in the VITI was higher (25%), overall, in the morning than the afternoon (F_(1,43)_ = 5.4, *P*<0.05) but there was no interaction with genotype (Fig. 6D).

## Discussion

Mice lacking functional NK1 receptors are capable of learning the 5-CSRTT, as has been reported for their background strain (C57BL/6x129Sv: [25]). However, NK1R-/- mice needed more training sessions overall and expressed deficits in their cognitive performance that resembled those found in ADHD patients [see: 2]. This combination of findings is similar to that from studies of ADHD patients in whom a deficit in executive function, rather than primary learning disability, explains their impaired performance [26].

### Omissions

There was a greater incidence of *omissions* (an index of inattentiveness) in NK1R-/- mice than their wildtypes in all stages of this study. This was particularly evident during the early stages of training. The convergence of the performance of the two genotypes at Stage 6, suggests that NK1R-/- mice adapt slowly to step-changes in stimulus parameters, especially prolongation of the ITI (see Table 2: Materials and Methods). The resurgence of greater *% omissions* in NK1R-/- mice when the inter-trial interval was subsequently adjusted in the LITI and VITI supports this proposal.

**Table 2:** Schedule for stimulus parameters during Stages 1 to 6 (training) and testing in the 5-CSRTT.

***Pretraining***	Habituation to apparatus			All apparatus lights switched on	
	Reward from magazine			Reward continuously available from magazine	
	Stimulus holes illuminated constantly			All stimulus holes illuminated: reward offered on nose-poke through any hole	
***Training***	Only one (of five) stimulus holes is illuminated in any trial. A nose-poke into this hole triggers reward				
	**Stages**	**Parameters used**			**Progression criteria**
		SD† (s)	LH† (s)	ITI† (s)	
	**1**	30	30	2	>30 correct trials for 2 consecutive days
	**2**	20	20	2	Unchanged
	**3**	10	10	5	>50 correct trials for 2 consecutive days
	**4**	5	5	5	>50 correct,>75% accuracy,<25% Omission errors for 2 consecutive days
	**5**	2.5	5	5	Unchanged
	**6**	1.8	5	5	Unchanged
***Tests***	**Long ITI (LITI)**	1	5	7	N/A
	**Variable ITI (VITI)**	1.8	5	2, 5, 10, 15	N/A
***Drug testing***	Mice were tested with no treatment (NI-1) and then retested with neither vehicle nor drug treatment (NI-2), or after injection of either vehicle or d-amphetamine (0.3 or 1 mg/kg i.p). Mice experienced each test condition once, only. The sequence was semi-randomised (Latin-square) with a one-week interval between each test.				

† SD: stimulus duration, LH: limited hold, ITI: inter-trial interval.

The higher *% omissions* in NK1R-/- mice, during training, is unlikely to be due to difficulties with task acquisition because *accuracy* and *latency to correct response* did not differ from wildtypes. These findings, coupled with the locomotor hyperactivity of NK1R-/- mice [8], [11], also rule out problems with motor function and visual discrimination. Greater *% omissions* in NK1R-/- mice could be explained by a reduction in their motivation to respond, which would be consistent with their greater *latency to collect the reward*. However, there was no genotype difference in *latency to correct response* (an alternative index of motivation) during training or the LITI. Furthermore, the two genotypes did not differ in their *latency to collect the reward* in the VITI. All these findings offer evidence against any influence of genotype on animals' motivation to respond in this test. The most likely (and usual) interpretation of a higher incidence of *% omissions* is that they reflect ‘inattentiveness’, which is a core feature of ADHD.

Inattentiveness has been attributed to abnormal (deficient or excessive) phasic release of norepinephrine in the prefrontal cortex. The optimal phasic response depends on background tonic activity [27]). Norepinephrine transmission has also been linked with attention in the 5-CSRTT [28], especially when the stimulus/reward contingency is altered [29]. It follows that the greater tonic release of norepinephrine in corticostriatal brain regions of NK1R-/- mice [9], [11] could contribute to their inattentiveness.

*d-*Amphetamine had no appreciable effect on *% omissions* of NK1R-/- and even tended to increase it in wildtypes, as in outbred rats [30]. This exacerbation of inattentiveness is unlikely to be due to any anorectic effect of *d*-amphetamine because there were no consistent changes in *latency to correct response* or *latency to collect the reward* in either the LITI or the VITI and the effects of *d*-amphetamine on these measures did not differ in the two genotypes.

If excessive norepinephrine transmission in NK1R-/- mice underlies their inattentiveness, then it is not surprising that *d*-amphetamine, a potent norepinephrine releasing-agent, did not diminish their inattentiveness. *d*-Amphetamine would be expected to be beneficial only in subjects with a deficit in norepinephrine transmission in the prefrontal cortex. This proposal is supported by reports that *d*-amphetamine reduces the inattentiveness of the Spontaneously Hypertensive Rat (SHR) [31], which is the benchmark rodent model of ADHD and has a lower concentration of extracellular norepinephrine in the prefrontal cortex than their control strain [32]. Therefore, a lack of a therapeutic response to *d*-amphetamine might serve as a marker for patients with polymorphism(s) in the region of the *tacr1* gene. The NK1R-/- mouse model of ADHD predicts that such patients would benefit from treatments that would augment, or mimic, neurotransmission governed by activation of NK1R.

### Perseveration

*Perseveration* of NK1R-/- mice was consistently greater than that of wildtypes during training in the LITI and VITI. This behavior is not a diagnostic feature in ADHD but is a common co-morbid complication [33], [34].

A potentially important caveat is that saline injection increased *perseveration* of NK1R-/- in the LITI but reduced that of wildtypes. This suggests that provocation of *perseveration* by stress is prevented by activation of NK1R. When *d*-amphetamine, rather than saline, was injected there was a dose-dependent attenuation of *perseveration* of NK1R-/- mice. Injection of *d*-amphetamine did not cause any further reduction in *perseveration* in wildtypes, possibly because of a floor effect. A similar, albeit less clear-cut, pattern of changes in *perseveration* emerged in the VITI.

*Perseveration* is typically linked with a deficit in dopaminergic transmission in the neuronal circuit linking the ventral tegmental area (VTA), the prefrontal cortex, nucleus accumbens and dorsomedial striatum [35], [36], [37]. The reduced extracellular dopamine in the prefrontal cortex of NK1R-/- mice [10] is consistent with this proposal. Stress increases release of dopamine in corticostriatal regions [38] and so would be predicted to reduce *perseveration* in wildtypes, as was found here.

Stress also increases release of substance P [39] and activation of NK1R is essential for the dopamine response to stress [40]. It follows that a lack of functional receptors in NK1R-/- mice would blunt the dopamine response to injection-stress and so prevent inhibition of *perseveration*. This leads to the possibility that *d-*amphetamine mimics the effects of stress by triggering impulse-independent release of dopamine in the terminal field [41]). Suppression of *perseveration* by stress is consistent with evidence that behavioral control is most impaired in ADHD patients with a blunted (cortisol) response to stress [42].

On the basis of these findings, we infer that relief of *perseveration* by *d*-amphetamine does not require functional NK1R.

### Premature responses

A higher incidence of *premature responses* (impulsivity) in NK1R-/- mice was evident during the VITI. Impulsivity has long been associated with abnormal serotonergic transmission, which disrupts functional coupling of corticostriatal regions [43], [44]. *Premature responding* in the 5-CSRTT correlates positively with serotonin efflux in the prefrontal cortex [45] and is induced by activation of serotonin_2A_ or serotonin_2C_ receptors [46], [47]. Although other monoamines can influence impulsivity [*e.g.,* [45], [48], [49], [50]], the greater serotonin release in the prefrontal cortex of NK1R-/- mice, compared with the wildtypes [12], is consistent with their impulsivity.

A feature shared by the transition from Stage 2 to Stage 3 of training (when *premature responding* was increased in both genotypes) and the VITI test is that prolongation of the ITI is unpredictable in both cases (see Table 2). This suggests that animals' response control is influenced by their anticipation of the light signal and perception of the time that has passed since the start of the trial, as has been found in ADHD patients [51], [52], [53]. Serotonin has a key role in interval timing [54]: the greater release of this transmitter in NK1R-/- mice [12] might aggravate impulsivity by disrupting their perception of the passage of time.

*d*-Amphetamine did not diminish *premature responses* in either the LITI or the VITI: the higher dose even exacerbated this behavior in the latter test. Because serotonergic transmission is increased in NK1R-/- mice at baseline [12], a further increase in serotonin release following administration of a high dose of *d*-amphetamine [41], [55] would be expected to exacerbate impulsivity.

There are inconsistent reports on the effects of *d*-amphetamine on *premature responses* when (outbred) rats and mice are studied in the 5-CSRTT: both a reduction (LITI: [30]; LITI or VITI: [56]) and an increase [57], [58], [59], [60] have been reported. Reasons for these disparate findings are not known, but *d-*amphetamine does have beneficial effects in other measures of impulsivity in rodents (*e.g.,* ‘delay-discounting’: [61]). This could be because different test procedures probe different types of impulsivity [18], which will have different neurobiological substrates.

*d*-Amphetamine also reduces impulsivity in the SHR model of ADHD [31]. Although, to the best of our knowledge, the SHR has not been tested in the 5-CSRTT, it is striking that basal serotonergic release is not increased in their prefrontal cortex [62]. Evidence suggests that insufficient, as well as excessive, serotonin transmission can provoke impulsivity (see: [48]) and so it is possible that a *d*-amphetamine-induced increase in serotonin release improves response control in the SHR but not the NK1R-/- mouse. Furthermore, the lack of any improvement in the NK1R-/- mouse suggests that the response to *d*-amphetamine normally recruits functional NK1R. If so, relief of impulsivity in ADHD patients with impaired NK1R function would need a treatment that either augments activation of these receptors or mimics the downstream response.

### Circadian influences

The incidence of both *omissions* and *premature responses* in NK1R-/- mice depended on time of day. *Omissions* in the VITI were slightly lower in the afternoon but the lack of interaction between genotype and time of day means that NK1R do not influence this circadian change. Nevertheless, it is interesting that inattentiveness in ADHD is more pronounced in patients who orient their behavior towards the evening (‘owls’ or ‘evening types’: [22]), especially in the Predominantly Inattentive subgroup.

By contrast, genotype did affect a circadian influence on *premature responding* because the transient increase during Stage 3 of training did not occur in NK1R-/- mice trained in the morning. A similar pattern emerged with the LITI. Circadian fluctuation of impulsivity has been found in humans, also [63]. The lack of any effect of time of day on *premature responses* in the VITI could suggest that exacerbation of impulsivity by unpredictable, prolonged ITIs masks any circadian influences on this behavior.

NK1R are abundantly expressed in the intergeniculate leaflet of the mouse and, to a lesser extent, by neurons along the dorsolateral border region of the suprachiasmatic nucleus [64]. Both areas are strongly linked with circadian rhythms and their entrainment. Abnormal neurotransmission at either of these sites could disrupt a circadian regulation of *premature responses* in NK1R-/- mice. Whether or not this is correct, our findings suggest that time of day might be a key variable in studies of ADHD patients and that the effect of an interaction between NK1R function and circadian rhythms on response control merits further investigation.

### Conclusion

NK1R-/- mice display deficits in cognitive performance and response control that resemble diagnostic features of ADHD: namely, inattentiveness, impulsivity and perseveration. Injection stress increased *perseveration* in NK1R-/- mice and this increase was prevented by *d*-amphetamine, which otherwise did not diminish the performance deficits in this genotype. The incidence of *omissions* (VITI) and *premature responses* (LITI) were influenced by time of day. Moreover, the incidence of the latter behavior depended on an interaction between genotype and time of day, suggesting coupling between NK1R activation and neuronal circuits that govern circadian rhythms and response control. Collectively, our findings consolidate the NK1R-/- mouse as a model of ADHD, possibly of the Predominantly Inattentive subtype and further suggest that time of day, the test parameters, and stress are variables that could influence the outcome of translational studies.

## Materials and Methods

### Ethics Statement

These experiments were licensed under the Animals (Scientific Procedures) Act, 1986 (UK) and had local ethical approval at University College London and the University of Sussex.

### Animals

We used male wildtype and NK1R-/- mice (25–40 g and 6–8 weeks of age at the start of each experiment) from a colony based at UCL. Both genotypes derived from a 129/Sv *x* C57BL/6 genetic background, crossed with an outbred MF1 strain (Harlan OLAC, Bicester, UK), for one generation, many generations ago [7]. The facility was held at 21±2°C, 45±5% humidity, and a 12∶12 h light: dark cycle (lighting increased gradually from 07.00–08.00 h). The cages incorporated environmental enrichment and were cleaned twice a week (bedding: Litaspen Premiun (Lillico)). Water was freely available throughout the study, from standard water bottles with a nozzle that penetrated the cage lid. Access to food (2018 global Rodent Diet (Harlan)) was adjusted to stabilise each subject at 90% of free-feeding body weight. The mice were weighed every morning before training/testing in the 5-CSRTT.

In two separate experiments, using the same training/testing procedures, four mice (in each experiment) were taken, at random, from three breeding pairs for each genotype. These groups of four mice were housed together such that every ‘home cage’ contained four wildtype or four NK1R-/- mice. Two mice of each genotype from each cage were trained and tested in the morning while the remainder were trained and tested in the afternoon. These cage groups were maintained throughout the experiments. One mouse from each cohort died before the end of the experiment, leaving N = 11 for the remainder of the experiment.

### Apparatus (5-CSRTT)

The apparatus comprised four mouse operant chambers, each housed within a ventilated sound-attenuating box (Med Associates, St. Albans, VT, USA). The rear wall of the chamber was curved and incorporated five equally-spaced apertures. Inside each of these was a stimulus light, used to illuminate the hole, and an infrared detector for monitoring nose-pokes by the mouse. A hole in the front wall provided access to a magazine that delivered a liquid reward (0.01 mL of 30% condensed milk solution), which was signalled by illumination of the magazine. Head entries into the magazine, to collect the reward, were scored following interception of an infrared photo-cell beam. A house-light, to illuminate the test chamber, was mounted above the magazine. The presentation of the light stimuli and recording of the animals' responses were controlled by a Smart Ctrl Package 8IN/16OUT with an additional interface by MED-PC for Windows (Med Associates, St. Albans, VT, USA).

### 5-Choice Serial Reaction-Time Task

Subjects were consistently brought into the laboratory (Monday to Friday) at 09.30 h and were trained/tested, as described below, either between 10.00–12.00 h or 13.00–15.00 h. This enabled us to study circadian influences on behavior. To eliminate any influence of ‘cage effect’ on behavior, half the mice in each cage were trained and tested in the morning: the remainder were trained and tested in the afternoon. On any given experimental day, the same researchers were responsible for all the procedures in both the morning and afternoon to ensure that variables such as handling and auditory/olfactory signals were balanced across all subjects. After being tested in the LITI and the VITI, the first cohort of mice was used to study the effects of *d-*amphetamine on behavior. Findings from a study of the effects of a different drug challenge in the 5-CSRTT, using the second cohort, will be the subject of a separate report.

#### Habituation

Mice were placed in the 5-CSRTT chamber for 30 min, once-daily for three consecutive days. All the lights in the apparatus (house light, the five apertures and food magazine) remained switched on. To receive the liquid reward, mice were required to nose-poke into the magazine. The reward was given on a continuous reinforcement schedule and was available for 10 s after each nose-poke, after which the dipper was retracted and refilled. The number of head-entries into the magazine and number of reinforcers earned was recorded on-line.

After three days of habituation, the mice were trained on a non-spatial schedule. Now, only the lights in the five apertures were switched on, but every nose-poke (into any hole) was rewarded by delivery of milk solution to the magazine. Before progressing to training in the 5-CSRTT, the mice were required to earn more than 50 reinforcers, for two consecutive days, over a maximum of 10 sessions.

#### Training

After the habituation phase, the mice were trained to carry out the 5-CSRTT. The difficulty of the task increased progressively from Stages 1 to 6. To graduate from one stage to the next, each animal's behavior had to stabilise at specific performance criteria (Table 2). Training/testing was carried out once-daily, five days a week.

At the beginning of each session, the house light and the magazine light (the latter signalling delivery of the reward) were switched on. The first trial was started by a nose-poke into the magazine to collect the reward. After an inter-trial interval (ITI) of fixed duration (determined by the stage of the training: see Table 2), the stimulus light in one of the five apertures was switched on, again for a fixed duration (‘Stimulus Duration’, SD). The animal was required to nose-poke into the illuminated hole within a fixed time interval after the onset of the stimulus (‘Limited Hold’, LH). The sequence for illuminating each of the five holes was randomised. A correct response was rewarded by delivery of milk to the magazine, which was signalled by the magazine light (as above). The next trial of the session was initiated by the mouse collecting the reward.

If the animal did not respond correctly (e.g., nose-poking into a non-illuminated hole), or failed to nose-poke within the allowed time (omission error), or responded prematurely (i.e., nose-poke into the holes during the ITI: i.e., before the onset of the stimulus), the house light was extinguished for 5 s (‘Time Out’, TO). A nose-poke into any of the five holes during this period restarted the TO. At the end of the TO, the mouse was allowed to start the next trial with an unrewarded nose-poke into the illuminated magazine. Each session finished after 30 min or completion of 100 trials, whichever occurred first.

#### Performance during long ITI (LITI) or variable ITI (VITI) test: effect of *d*-amphetamine

After stable performance at Stage 6 for at least 7 consecutive days (baseline), treatment-naïve mice (NI-1) were tested in a session in which the ITI was prolonged (7 s) but remained constant (long ITI': ‘LITI’). The stimulus duration was reduced to 1 s (*cf* [19]) and the duration of the session was increased to 45 min. Starting one week later, the mice were retested, at once-weekly intervals, with the same LITI test but, during these sessions, they were tested 30 min after an intraperitoneal (i.p.) injection of saline (10 ml/kg) or *d*-amphetamine (0.3 or 1 mg/kg), or no injection of either saline or drug (‘NI-2’). These doses of *d*-amphetamine were chosen because they are reported to influence mouse cognition behavior at doses equivalent to those used to treat ADHD [65] and are lower than those that increase locomotor activity [66]. The second, injection-free challenge (NI-2) was to control for improvement in animals' performance, as a consequence of rehearsing the task, and was embedded randomly (Latin square) within the series of once-weekly assessments the effects of saline or *d*-amphetamine. Every mouse experienced each test condition, once only. During the intervening week, animals were subject to once-daily sessions at Stage 6 to ensure that their behavior was restored to the stable baseline before the next test. This series of tests was then repeated, substituting a variable ITI (VITI: see Table 2) for the LITI. The VITI could be any one of four alternatives (2, 5, 10 or 15 s), delivered on a random schedule.

In a second cohort of mice (‘Cohort 2’), the procedures were the same with the exception that uninjected mice (N1-1) were tested with the VITI before the LITI, so as to counterbalance the sequence experienced by Cohort 1, before going on to test the effects of a different compound at weekly intervals (not reported here).

### Behavioral scoring

The following performance variables in the 5-CSRTT training and tests were scored and stored online:

- ***Total number of sessions required to pass the training phase***: the sum of all the sessions completed over Training Stages 1–6.
- ***Total number of trials completed in each test session:*** total correct responses + total incorrect responses + total omissions during the LITI or VITI test.
- ***% Accuracy*** **:** [correct responses/(correct + incorrect responses)]×100.
- ***% Omissions:*** [total omissions/(correct + incorrect responses + omissions)]×100.
- ***% Premature responses*** **:** [premature responses/(correct + incorrect + omissions + premature responses)]×100.
- ***Latency to correct response:*** latency to nose-poke into the correct hole after the onset of stimulus.
- ***Latency to collect the reward (reach the magazine):*** latency to collect the reinforcer after a correct response.
- ***Perseveration score*** **:** total number of responses into the same, correct hole during the interval between a correct response and collection of the reinforcer.

### Statistical analysis

Statistical analyses were carried out on the raw data, log_10_-transformed, (score + 1)log_10_-transformed or square-root-transformed data, according to whichever produced the least significant value in the Levene's test. We pooled data from the two cohorts if the influence of the factor(s) of interest on behavior did not differ, as in the training sessions and the LITI. In the VITI, there were differences in the incidence of certain aspects of behavior of the two cohorts (Table 1). However, there was no interaction between the factor ‘Cohort’ and either ‘genotype’ or ‘time of day’ (*i.e*., the influence of neither ‘genotype’ nor ‘time of day’ depended on cohort) and so we again pooled the data when looking for main effects of these two factors on behavior. The results of all the statistical comparisons, for all parts of this study, are given in Supporting Information (Tables S1, S2, S3, S4, S5, S6, S7 and S8).

Raw or transformed data were first analyzed using 3-way repeated measures ANOVA (SPSS PC^+^) with ‘cohort’, ‘genotype’ and ‘time of day’ as between-subjects factors, and ‘training stage’ or ‘test treatment’ as within-subjects factors. In tests of repeated measures, the Greenhouse-Geisser ‘ε’ correction was applied routinely to data sets that showed statistical significance in Mauchley’s sphericity test. A significant effect of one of the main factors, or a relevant interaction between them, was used as the criterion for progressing to 2-way or 1-way ANOVA with *post hoc* comparisons of the data (LSD test or matched-pair and/or independent-samples *t*-test, or the non-parametric Mann-Whitney U-test, as appropriate). Statistical significance was set at *P*<0.05.

## Supporting Information

Table S1 Statistical comparisons of behaviour in NK1R-/- and wildtype mice during training stages 1–6.
(DOC)

Table S2 Number of training sessions needed to match the baseline criteria for testing.
(DOC)

Table S3 Statistical analysis of the effect of genotype and time of day on behavior of uninjected mice, tested for the first time (NI-1), with a long ITI (LITI).
(DOC)

Table S4 Statistical analysis of the effect of genotype and time of day on behavior of uninjected mice, tested for the first time (NI-1), with a variable ITI (VITI).
(DOC)

Table S5 Statistical comparisons of behavior during the LITI: NI-2 versus vehicle-injected mice.
(DOC)

Table S6 Statistical comparisons of behavior in vehicle- and d amphetamine (0.3 mg/kg or 1 mg/kg (i.p.)) treated mice in the LITI.
(DOC)

Table S7 Statistical comparisons of behavior during the VITI: NI-2 versus vehicle-injected mice.
(DOC)

Table S8 Statistical comparisons of behavior in vehicle- and d amphetamine (0.3 mg/kg or 1 mg/kg (i.p.)) treated mice in the VITI.
(DOC)
