# Supplementary material for: Performance Deficits of NK1 Receptor Knockout Mice in the 5-Choice Serial Reaction-Time Task: Effects of d-Amphetamine, Stress and Time of Day
Source: PLoS One. 2011 Mar 7;6(3):e17586. doi: 10.1371/journal.pone.0017586 (PMC3049786; doi:10.1371/journal.pone.0017586)
Supplement: Table S1 — Statistical comparisons of behaviour in NK1R-/- and wildtype mice during training stages 1–6. (DOC) [file pone.0017586.s001.doc]

| **Measure** | **Genotype** | **Time of day** | **Genotype *x* Time of day** |
| --- | --- | --- | --- |
| *% Accuracy* | F_(1,39)_ = 0.3 | F_(1,39)_ = 2.5 | F_(1,39)_ = 0.8 |
|  | NS | NS | NS |
| *% Omissions* | F_(1,39)_ = 8.2 | F_(1,39)_ = 2.1 | F _(1,39)_ = 0.2 |
|  | *P* = 0.01 | NS | NS |
| *% Premature responses* | F_(1,43)_ = 11.5 | F_(1,43)_ = 16.5 | F_(1,43)_ = 2.0 |
|  | *P* < 0.001 | *P* < 0.001 | NS |
| *Latency to correct response* | F_(1,39)_ = 0.8 | F_(1,39)_ = 1.6 | F_(1,39)_ = 2.6 |
|  | NS | NS | NS |
| *Latency to collect the reward* | F_(1,39)_ = 22.8 | F _(1,39)_ = 0.1 | F_(1,39)_ = 2.9 |
|  | *P* < 0.001 | NS | NS |
| *Perseveration* | F_(1,39)_ = 23.3 | F_(1,39)_ = 1.6 | F_(1,39)_ = 0.3 |
|  | *P* < 0.001 | NS | NS |
| NS: P > 0.05 (not significant) | | | |
