# Supplementary material for: Performance Deficits of NK1 Receptor Knockout Mice in the 5-Choice Serial Reaction-Time Task: Effects of d-Amphetamine, Stress and Time of Day
Source: PLoS One. 2011 Mar 7;6(3):e17586. doi: 10.1371/journal.pone.0017586 (PMC3049786; doi:10.1371/journal.pone.0017586)
Supplement: Table S2 — Number of training sessions needed to match the baseline criteria for testing. (DOC) [file pone.0017586.s002.doc]

| **Genotype** | **Time of day** | **Genotype *x* Time of day** |
| --- | --- | --- |
| F_(1,43)_ = 4.1, *P* < 0.05 | F_(1,43)_ = 5.7, *P* < 0.05 | F_(1,43)_ = 1.4, NS |
| NS: P > 0.05 (not significant) | | |
