# Supplementary material for: Performance Deficits of NK1 Receptor Knockout Mice in the 5-Choice Serial Reaction-Time Task: Effects of d-Amphetamine, Stress and Time of Day
Source: PLoS One. 2011 Mar 7;6(3):e17586. doi: 10.1371/journal.pone.0017586 (PMC3049786; doi:10.1371/journal.pone.0017586)
Supplement: Table S5 — Statistical comparisons of behavior during the LITI: NI-2 versus vehicle-injected mice. (DOC) [file pone.0017586.s005.doc]

| **Measure** | **Genotype** | **Time of day** | **Genotype *x* Time of day** |
| --- | --- | --- | --- |
| *% Accuracy* | F_(1,22)_ = 2.3 | F_(1, 22)_ = 0.8 | F _(1, 22)_ < 0.001 |
|  | NS | NS | NS |
| *% Omissions* | F_(1,22)_ = 5.1 | F_(1, 22)_ = 1.8 | F _(1, 22)_ = 4.7 |
|  | *P* < 0.05 | NS | *P* < 0.05 |
| *% Premature responses* | F_(1,22)_ = 0.1, | F_(1, 22)_ = 2.2 | F_(1, 22)_ = 0.3 |
|  | NS | NS | NS |
| *Latency to correct response* | F_(1, 22)_ = 3.6 | F_(1, 22)_ = 1.9 | F_(1, 22)_ = 0.2 |
|  | NS | NS | NS |
| *Latency to collect the reward* | F_(1,22)_ = 6.9 | F _(1, 22)_ = 2.3 | F_(1, 22)_ = 7.1 |
|  | *P* < 0.05 | NS | *P* < 0.05 |
| *Perseveration* | F_(1,22)_ = 3.8 | F_(1, 22)_ = 1.7 | F _(1, 22)_ = 9.8 |
|  | NS | NS | *P* < 0.01 |
| NS: P > 0.05 (not significant) | | | |
