# Supplementary material for: Performance Deficits of NK1 Receptor Knockout Mice in the 5-Choice Serial Reaction-Time Task: Effects of d-Amphetamine, Stress and Time of Day
Source: PLoS One. 2011 Mar 7;6(3):e17586. doi: 10.1371/journal.pone.0017586 (PMC3049786; doi:10.1371/journal.pone.0017586)
Supplement: Table S8 — Statistical comparisons of behavior in vehicle- and d amphetamine (0.3 mg/kg or 1 mg/kg (i.p.)) treated mice in the VITI. (DOC) [file pone.0017586.s008.doc]

| **Measure** | **Genotype** | **Time of day** | **Genotype x Time of day** |
| --- | --- | --- | --- |
| *% Accuracy* | F _(1, 21)_ = 6.2 | F _(2, 42)_ = 3.6 | F _(2, 42)_ = 0.7 |
|  | *P* < 0.05 | *P* < 0.05 | NS |
| *% Omissions* | F _(1, 21)_ = 2.7 | F _(2, 42)_ = 0.4 | F _(2, 42)_ = 1.4 |
|  | NS | NS | NS |
| *% Premature responses* | F _(1, 21)_ = 3.5 | F _(2, 42)_ = 3.6 | F _(2, 42)_ = 2.3 |
|  | NS | *P* < 0.05 | NS |
| *Latency to correct response* | F _(1, 21)_ = 8.1 | F _(2, 33)_ = 4.1 | F _(2, 33)_ = 0.9 |
|  | *P* < 0.01 | *P* < 0.05 | NS |
| *Latency to collect the reward* | F _(1, 21)_ = 9.9 | F _(1, 30)_ = 7.9 | F _(1, 30)_ = 0.6 |
|  | *P* < 0.01 | *P* = 0.001 | NS |
| *Perseveration* | F _(1, 21)_ = 5.5 | F _(2, 42)_ = 1.7 | F _(2, 42)_ = 0.2 |
|  | *P* < 0.05 | NS | NS |
| NS: P > 0.05 (not significant) | | | |
